# Precision Design of Antimicrobial Surfaces

**DOI:** 10.3389/fmedt.2021.640929

**Published:** 2021-02-16

**Authors:** Declan C. Mullen, Xing Wan, Timo M. Takala, Per E. Saris, V. M. Moreira

**Affiliations:** ^1^Strathclyde Institute of Pharmacy and Biomedical Sciences, University of Strathclyde, Glasgow, United Kingdom; ^2^Department of Microbiology, Faculty of Agriculture and Forestry, University of Helsinki, Helsinki, Finland; ^3^Laboratory of Pharmaceutical Chemistry, Faculty of Pharmacy, University of Coimbra, Coimbra, Portugal; ^4^Centre for Neuroscience and Cell Biology, University of Coimbra, Coimbra, Portugal

**Keywords:** surface, antimicrobial, biofilm, interface, bacteria

## Abstract

The overall expectation from an antimicrobial surface has been high considering the need for efficiency in preventing the attachment and growth of pathogenic microbes, durability, safety to both humans and environment as well as cost-effectiveness. To date, antimicrobial surface design has been mostly conducted liberally, without rigorous consideration of establishing robust structure-activity relationships for each design strategy or of the use intended for a specific antimicrobial material. However, the variability among the domain bacteria, which is the most diverse of all, alongside the highly dynamic nature of the bacteria-surface interface have taught us that the likelihood of finding universal antimicrobial surfaces is low. In this perspective we discuss some of the current hurdles faced by research in this promising field, emphasizing the relevance and complexity of probing the bacteria-surface interface, and explain why we feel it would greatly benefit from a more streamlined *ad-hoc* approach.

## Antimicrobial Surfaces—Why Are They so Important?

Interest in the development of antimicrobial surfaces has escalated in the last two decades. Literature searches on the Web of Science reveal impressive 3- and 6-fold increases in the number of original and review articles as well as in patents devoted to antimicrobial surfaces, from 2000 to 2010 and from 2000 to 2020, respectively. Patents alone account for 29% of the 42,691 publication universe under the keyword “antimicrobial surface.”

This interest in antimicrobial surfaces goes hand-in-hand with the 2012–2022 explosion in the global market for nanoengineered surfaces (NES) where the building sector heads the expected million USD revenues, followed by electronics and the biomedical sector (1). Within the latter, the sub-sectors of anti-bacterial sterilization and anti-biofouling radically evolved from having a negligible value in 2012 to an expected value of 106.4 and 51.7 billion USD by 2022, respectively, with an estimated total Compound Annual Growth Rate (CAGR) of 127.5% in this period.

Antimicrobial surfaces are needed to prevent the growth and spread of infectious microbes on a plethora of materials that routinely serve humans. They have become ubiquitous and indispensable in extending the shelf-life of both consumer and industrial goods as well as in reducing health risks across a wide range of sectors including health, food packaging, furniture, textiles, and the building and shipping industries (2–5). The outstanding impact of antimicrobial surfaces on boosting future technologies is predicted in the design of self-driving cars, for instance, where they will help to reduce the maintenance and downtime of key parts (6).

The need to build physical barriers between humans and infectious agents to prevent their spread within our community by contact has very recently been evidenced by the global pandemic caused by SARS-CoV-2. Although extensive efforts have been directed toward the design of surfaces to target bacteria, little has been done to find those that efficiently kill and/or repel viruses, with the first steps toward understanding the method and duration of their surface adherence currently taking place (5, 7). At present, the remarkable evolution of community-disseminated super-resistant bacteria, alongside the scarcity of new antibacterial drugs to have reached the market in the past decades (8), represents a latent menace that threatens to cause the next global health crisis.

Overall, the expectations from an antimicrobial surface have been high. They should efficiently prevent the attachment and growth of pathogenic microbes indiscriminately thus limiting their spread by contact, be durable, harmless to human health and to the environment and cost-effective. However, can one single surface meet such a highly demanding wish list? Is the surface development process conveniently streamlined to ensure that the upcoming years will witness significant advances in the biomedical field?

## Antimicrobial Surfaces That Leach

By far the most straightforward strategy to design surfaces that target bacteria remains the incorporation, by physical adsorption, of an antimicrobial agent onto a polymeric matrix (2–5). Such surfaces are deemed leaching, i.e., they kill bacteria upon release of the antimicrobial agent over time. Despite being effective, leaching surfaces will eventually become inactivated once the antimicrobial agent has been exhausted and cannot therefore be regarded as long-lasting solutions. In addition, they are only as good as the agent they release, i.e., there is a limited number of antimicrobial agents that can be used due to stringent regulations.

Although the mode of action of leaching surfaces is easily ascribed to the respective agent they release, the exact load of antimicrobial agent comprised by the surface can be hard to accurately quantify, and the environmental impact of the leaching process is of concern (9). Metals and metal salts including silver, copper, zinc, and titanium dioxide are the most commonly used. They are known to act by inducing bacterial membrane disruption and oxidative stress. Long-term toxicity associated with exposure to silver is not yet fully established in humans, but its ecotoxicity is well-documented (10). Quaternary ammonium compounds (QACs), bearing permanent positive charges that disrupt bacterial membranes, lack sufficient efficiency and are prone to development of bacterial resistance (11, 12). In a similar fashion, bacteriostatic triclosan was banned over toxicity to both humans and the environment (13).

Natural antimicrobial peptides (AMPs), both bacterial and human, have also been under investigation (14). Among the diverse mechanisms of action known for AMPs, their net charges may allow for interaction with cell membranes while hydrophobic regions can maneuver into the phospholipid bilayers and in some instances result in pore formation and leakage of cell components (15). However, as AMPs are part of the innate immune system of all multicellular organisms, the potential for resistance development cannot be overlooked, particularly if human AMPs are employed. AMPs can also be chemically grafted at the surface of polymers (14, 16–22) and in this case the leaching ability will depend upon the coupling method selected which will determine the stability of the chemical bond established. Amides are among the strongest chemical bonds whereas esterification and silanization will afford less stable bonds. A quick agar plate test is usually sufficient to rule out this leaching effect. AMPs are chemically complex molecules and therefore any translation of their outstanding antimicrobial properties will likely rely on the development of simplified synthetic counterparts.

Nonetheless, antimicrobial surfaces that leach have been successfully translated into very useful practical applications. For instance, despite the fact that roughly 1/3 of the silver present in conventional wound-dressings leaches out and becomes black due to oxidation hindering visualization of the healing process, silver-based dressings are a mainstay (23) among antimicrobial dressings, a market valued at 9.16 billion USD in 2014 and expected to exceed more than 23 billion by 2024 (24).

## The Need to Explore the Interface With Bacteria

The early 90's realization that bacteria exist in nature as biofilms as opposed to single entities and the extensive knowledge of bacterial behavior gathered thereafter (25, 26), have impacted the paradigm of antimicrobial surface design. Biofilms are very seldom eradicated by leaching antimicrobial agents alone due to the presence of the sheltering extracellular matrix. One of the best depictions of this behavior is provided by *B. subtilis* biofilms (27) which are more non-water-wetting than Teflon, presenting extreme impenetrability to liquid antimicrobials and gases.

Intensive research into the physico-chemical mechanisms specifically involved in bacterial adhesion onto surfaces has been underway (28–32) in the hope of finding key events that can be targeted for limiting early biofilm establishment. In this regard, a dissection of the interactions occurring at the interface of antimicrobial surfaces and the outermost external components of bacterial cells has become crucial in order to explain how surfaces can either kill or repel bacteria (or both) directly upon contact. Such explorations have often been complemented by computational models to predict bacterial attachment (33, 34). These surfaces are referred to as contact-active, and typically they are complex, either entailing a pattern at the surface or a random arrangement, yet their mode of action is independent of any leaching substance. They are usually perceived as potentially more ecofriendly if they are biodegradable, and more efficient if they can overcome clogging by dead bacteria and/or debris over time.

For instance, QACs and antibiofilm peptides have been immobilized at the surface of several polymers leading to contact-killing activity (35–39). The regular separation of both positive and negative charges along zwitterionic polymers successfully resulted in anti-fouling and bactericidal properties with self-cleaning capacity (40). Immobilized bacteriocins such as nisin on various abiotic surfaces can prevent the formation of biofilms, and this approach has been explored by the food industry (41, 42). More recently, small tricyclic diterpenoids covalently bound onto nanocellulose through stable amide bonds (43, 44) rendered contact-active anionic antimicrobial surfaces capable of limiting biofilm formation.

Although hard to characterize both experimentally and theoretically, the tentative modes of action of contact-active surfaces are supported from studies regarding the activity of biosurfactants (45) and the interactions of nanoparticles with bacteria (32, 46, 47). It is likely that the surfaces are perceived by bacteria as complex polymeric matrices, unevenly branched with hydrophobic and hydrophilic regions or net charges that can intercalate into bacterial external structures, bind to surface proteins or modulate their activity through ion chelation, and/or have the ability to extract lipopolysaccharides, ultimately causing cell death. Other mechanisms may include enzymatic degradation of cellular membrane components or disruption of eDNA as well as limitation of the nutrient reservoir (48, 49). The presence of photoinduced compounds bound at the surface to kill bacteria by generation of oxidative radical species following activation has also been reported (35).

Regardless of the approach, the chemistry at the surface is a key determinant of the activity. Topographical manipulation of surfaces alone, i.e., devoid of any concomitant chemical modification, can compromise bacterial adhesion and in particular settings result in a contact-killing effect (50, 51). However, the number of materials that will entail the specific topographical features needed for the activity is limited and this strategy lacks sufficient efficiency to be regarded as a self-standing solution.

## Why Are Bacteria Winning the day?

The cumulative experience from the last two decades of research has taught us that bacteria-surface interfaces are outstandingly dynamic and that the likelihood of being successful with a simple approach, either leaching or non-leaching, is low. Therefore, the combination of leaching and non-leaching actions on the same surface, i.e., mixed action surfaces, has been investigated (2–5). One extreme example depicts the combination of topographical manipulation with chemical functionalization and the inclusion of a lubricating layer of liquid to build a slippery liquid-infused porous surface (SLIPS) that was able to prevent the attachment of both Gram-positive and Gram-negative bacteria for a whole week (52).

Indeed, the domain bacteria is the most diverse of all and this makes it virtually impossible to fine-tune a surface to meet the specific requirements of each bacterial strain in terms of hydrodynamics, topography-induced cell ordering, air-entrapment, chemical gradients, physicochemical force fields or cell membrane deformation, among other factors. In addition, even though bacteria use their surface structures, such as fimbriae, pili, flagella, and S-layer for adhesion to surfaces, these structures may also prevent bacteria or their membranes from coming into close contact with antimicrobial surfaces.

Bacteria have different preferences for hydrophilic and hydrophobic surfaces (53) which could relate to differences in charges and/or composition of their bacterial membranes and the extracellular polymeric matrix (EPS) they produce en route to establishing biofilms. In general, hydrophobic surfaces gain greater biofilm formation (54). As in antimicrobial drug discovery, the outer membrane of Gram-negative bacteria is a strikingly differentiating factor. The presence of lipopolysaccharide O-antigen is reported to hamper adhesion of surfaces onto bacteria by neutralizing the negative charge usually carried by the supporting cell envelope (32). Moreover, bacteria are well-prepared to adapt and evolve to survive in the presence of external stress. Finally, compared to research settings which work with primarily monoculture biofilms in controlled environments, natural biofilms also frequently host other microbes as symbionts to establish polymicrobial communities, thus challenging the performance of antimicrobial surfaces when used in real settings.

As exemplified by contact-active surfaces, a plethora of different surface chemistries will work against bacteria through manipulations of net charge, hydrophobicity, topography, or other factors, yet to date there is no clear cut structure-activity relationships that can be inferred to guide future design efforts. This is largely due to the diversity of polymer substrates, antimicrobial agents and functionalization strategies currently portrayed in the literature, which are extremely broad and essentially random, hampering what should be a systematic approach. At least one study has applied combinatorial chemistry and high-throughput screening to identify a group of structurally related polymers that limit pathogenic bacterial adhesion at their surface (55). With this approach, it is possible to focus on a single polymer class and determine, with a higher level of precision, exactly which variations in chemistry afforded the best antimicrobial properties. With this information at hand, predictive computational models can be built (29), yet their robustness is likely to be at present modest in light of the extreme complexity in accurately depicting bacteria-surface interactions.

On the other hand, while the majority of available reports focuses on finding broad-action antimicrobial surfaces, the translational value of selectively targeting one specific bacteria type remains to be determined. Clues on how to design surfaces that discriminate between Gram-positive and Gram-negative bacteria as well as fungi are available from literature on microbe detection systems (30). The fact that the activity of cationic polymers can be modulated by buffer concentration is notable.

## Time to Consolidate to Step up to the Challenge

The intricacy of bacteria-surface interactions turns the idea of an universal antimicrobial surface into a chimera. We foresee that advancements in this field will come from focusing the design of antimicrobial surfaces on the very specific features required by its intended use. This precision design will entail a comprehensive knowledge of the microbes that need to be targeted as well as of the polymers that bear the most convenient properties for good performance in a particular setting. These should include biopolymers such as (nano)cellulose, silk, collagen, or alginate for the sake of sustainability.

To support this endeavor, a very wide range of experimental, computational, and theoretical approaches will be mandatory where knowledge of chemistry including computational chemistry, microbiology, membrane biophysics and bioinformatics is key. In addition, the development of antimicrobial surfaces would greatly benefit from a “design of experiments approach” to streamline the process for building robust structure-activity relationships.

Our ability to continue to explore bacteria-surface interactions will dictate how much we can say of specific modes of action for each surface at the atomic level. For instance, despite significant advances in molecular dynamics to study the mode of action of small AMPs (56, 57), extending these studies to the scale and complexity of a surface is still way beyond the limits of this technique. Proteomics, transcriptomics, and mutagenesis studies will continue to be essential techniques in deciphering the interactions of antimicrobial surfaces with bacteria.

Regardless of addressing the main mode of action, the most important thing is functionality, i.e., to find surfaces that work. How broad-acting, durable, biodegradable, or cytocompatible they need to be should be dictated by their final use. Therefore, the selection of suitable control materials and bioassays that address the complexity of single-cell and multispecies biofilms is of utmost importance. Surfaces should also be screened in combination with other techniques to target biofilms including, for instance, cold plasmas (58). Finally, however specific these insights may be for bacteria, we believe the strategy for surface design outlined herein will apply for other microbes including fungi and viruses, conveniently adapted to their particular biology.

## Data Availability Statement

The original contributions generated for the study are included in the article/supplementary material, further inquiries can be directed to the corresponding author/s.

## Author Contributions

DM, XW, TT, and PS carried out literature searches. VM compiled the manuscript. All authors contributed to the views expressed in the article, and critically helped to write and revise the document.

## Conflict of Interest

The authors declare that the research was conducted in the absence of any commercial or financial relationships that could be construed as a potential conflict of interest.

## References

[1] Oliver J. Bioinspired and Nanoengineered Surfaces: Technologies, Applications and Global Markets - AVM089A. (2013). Available at: http://www.bccresearch.com/market-research/advanced-materials/bioinspired-nanoengineered-surfaces-avm089a.html?tid=eGMwdiov (accessed March 16, 2016).

[2] Hasan J Crawford J Ivanova EP. Antibacterial surfaces: the quest for a new generation of biomaterials. Trends Biotechnol. (2013) 31:295–304. 10.1016/j.tibtech.2013.01.017 23434154

[3] Banerjee I Pangule RC Kane RS. Antifouling coatings: recent developments in the design of surface that prevent the fouling by proteins, bacteria, and marine organisms. Adv Mater. (2011) 23:690–718. 10.1002/adma.201001215 20886559

[4] Adlhart C Verran J Azevedo NF Olmez MM Gouveia I Melo LF. Surface modifications for antimicrobial effects in the healthcare setting: a critical overview. J Hosp Infect. (2018) 99:239–49. 10.1016/j.jhin.2018.01.018 29410096

[5] Cassidy SS Sanders DJ Wade J Parkin IP Carmalt CJ Smith AM. Antimicrobial surfaces: a need for stewardship? PLoS Pathog. (2020) 16:e1008880. 10.1371/journal.ppat.1008880 33057433 PMC7561179

[6] Bettenhausen C. Self-driving cars are coming. Chemical makers are racing to keep up. C&EN News. (2020) 98:27–33. Available online at: https://cen.acs.org/business/consumer-products/Self-driving-cars-coming-Chemical/98/i41

[7] van Doremalen N Bushmaker AT Morris DH Holbrook MG Gamble A Williamson BN. Aerosol and surface stability of SARS-CoV-2 as compared with SARS-CoV-1. N Engl J Med. (2020) 382:1564–67. 10.1056/NEJMc2004973 32182409 PMC7121658

[8] Plackett B. Why big pharma has abandoned antibiotics. Nature. (2020) 586:S50–2. 10.1038/d41586-020-02884-3

[9] Bruenke J Roschke I Agarwal S Riemann T Greiner A. Quantitative comparison of the antimicrobial efficiency of leaching versus nonleaching polymer materials. Macromol Biosci. (2016) 16:647–654. 10.1002/mabi.201500266 26806336

[10] Seltenrich N. Nanosilver: weighing the risks and benefits. Environ Health Perspect. (2012) 121:A220–25. 10.1289/ehp.121-a220 PMC3702006 23816826

[11] Gerba CP. Quaternary ammonium biocides: efficacy in application. Appl Environ Microbiol. (2015) 81:464–9. 10.1128/AEM.02633-14 25362069 PMC4277564

[12] Jennings MC Minbiole KPC Wuest WM. Quaternary ammonium compounds: an antimicrobial mainstay and platform for innovation to address bacterial resistance. ACS Infect Dis. (2015) 1:288–303. 10.1021/acsinfecdis.5b00047 27622819

[13] Weatherley LM Goose JA. Triclosan exposure, transformation, and human health effects. J Toxicol Environ Health B Crit Rev. (2017) 20:447–69. 10.1080/10937404.2017.1399306 29182464 PMC6126357

[14] Riool M de Breij A Drijfhout JW Nibbering PH Zaat SAJ. Antimicrobial peptides in biomedical device manufacturing. Front Chem. (2017) 5:63. 10.3389/fchem.2017.00063 28971093 PMC5609632

[15] Jenssen H Hamill P Hancock RE. Peptide antimicrobial agents. Clin Microbiol Rev. (2006) 19:491–511. 10.1128/CMR.00056-05 16847082 PMC1539102

[16] Costa F Carvalho IF Montelaro RC Gomes P Martins MCL. Covalent immobilization of antimicrobial peptides (AMPs) onto biomaterial surfaces. Acta Biomaterialia. (2011) 7:1431–40. 10.1016/j.actbio.2010.11.005 21056701

[17] Yala J-F Thebault P Héquet A Humblot V Pradier C-M Berjeau J-M. Elaboration of antibiofilm materials by chemical grafting of an antimicrobial peptide. Appl Microbiol Biotechnol. (2011) 89:623–34. 10.1007/s00253-010-2930-7 20949268

[18] Etayash H Norman L Thundat T Kaur K. Peptide-bacteria interactions using engineered surface-immobilized peptides from class IIa bacteriocins. Langmuir. (2013) 12:4048–56. 10.1021/la3041743 23445325

[19] Song DW Kim SH Kim HH Lee KH Ki CS Park YH. Multi-biofunction of antimicrobial peptide-immobilized silk fibroin nanofiber membrane: implications for wound healing. Acta Biomater. (2016) 39:146–55. 10.1016/j.actbio.2016.05.008 27163404

[20] Homaeigohar S Boccaccini AR. Antibacterial biohybrid nanofibers for wound dressings. Acta Biomater. (2020) 107:25–49. 10.1016/j.actbio.2020.02.022 32084600

[21] Yang X Liu W Xi G Wang M Liang B Shi Y. Fabricating antimicrobial peptide-immobilized starch sponges for hemorrhage control and antibacterial treatment. Carbohyd Polym. (2019) 222:115012. 10.1016/j.carbpol.2019.115012 31320069

[22] Wang X Mao J Chen Y Song D Gao Z Zhang G. Design of antibacterial biointerfaces by surface modification of poly (ε-caprolactone) with fusion protein containing hydrophobin and PA-1. Colloids Surf B Biointerfaces. (2017) 151:255–63. 10.1016/j.colsurfb.2016.12.019 28027492

[23] Leaper D. An overview of the evidence on the efficacy of silver dressings. The silver debate. J Wound Care. (2011) 20(Suppl. 2):8–14. 10.12968/jowc.2011.20.Sup2.8 29480747

[24] Data from: Wound Dressing Market Size Share & Trends Analysis Report by Product [Traditional Advanced (Moist Antimicrobial Interactive)] by Region Segment Forecasts 2012-2022. Grand View Research (2018) Available online at: https://www.grandviewresearch.com/industry-analysis/wound-dressing-market (access December 1, 2020).

[25] Flemming H-C Wingender J Szewzyk U Steinberg P Rice SR Kjelleberg S. Biofilms: an emergent form of bacterial life. Nat Rev Microbiol. (2016) 14:563–75. 10.1038/nrmicro.2016.94 27510863

[26] Koo H Allan RN Howlin RP Stoodley P Hall-Stoodley L. Targeting microbial biofilms: current and prospective therapeutic strategies. Nat Rev Microbiol. (2017) 15:740–55. 10.1038/nrmicro.2017.99 28944770 PMC5685531

[27] Epstein AK Pokroy B Seminara A Aizenberg J. Bacterial biofilm shows persistent resistance to liquid wetting and gas penetration. Proc Natl Acad Sci USA. (2011) 108:995–1000. 10.1073/pnas.1011033108 21191101 PMC3024672

[28] Tuson HH Weibel DB. Bacteria-surface interactions. Soft Matter. (2013) 9:4368–80. 10.1039/C3SM27705D 23930134 PMC3733390

[29] Mkulskis P Hook A Dundas AA Irvine D Sanni O Anderson D. Prediction of broad-spectrum pathogen attachment to coating materials for biomedical devices. ACS Appl Mater Interfaces. (2018) 10:139–49. 10.1021/acsami.7b14197 29191009 PMC7613461

[30] Yuan H Liu Z Liu L Lv F Wang Y Wang S. Cationic conjugated polymers for discrimination of microbial pathogens. Adv Mater. (2014) 26:4333–8. 10.1002/adma.201400636 24737340

[31] Feng ZV Gunsolus IL Qiu TA Hurley KR Nyberg LH Frew H. Impacts of gold nanoparticle charge and ligand type on surface binding and toxicity to Gram-negative and Gram-positive bacteria. Chem Sci. (2015) 6:5186–96. 10.1039/C5SC00792E 29449924 PMC5669217

[32] Beaussart A Beloin C Ghigo J-M Chapot-Chartier M-P Kulakauskas S Duval JFL. Probing the influence of cell surface polysaccharides on nanodendrimer binding to Gram-negative and Gram-positive bacteria using single-nanoparticle force spectroscopy. Nanoscale. (2018) 10:12743–53. 10.1039/C8NR01766B 29946619

[33] Chinnaraj SB Jayathilake PG Dawson J Ammar J Portoles J Jakubovics N. Modelling the combined effect of surface roughness and topography on bacterial attachment. J Materials Sci Technol. (2021) 81:151–61. 10.1016/j.jmst.2021.01.011

[34] Acemel RC Govantes F Cuetos A. Computer simulation study of early bacterial biofilm development. Sci Rep. (2018) 8:5340. 10.1038/s41598-018-23524-x 29593289 PMC5871757

[35] Kaur R Liu S. Antibacterial surface design – contact kill. Progress Surf Sci. (2016) 91:136–53. 10.1016/j.progsurf.2016.09.001

[36] Siedenbiedel F Tiller JC. Antimicrobial polymers in solution and on surfaces: overview and functional principles. Polymers. (2012) 4:46–71. 10.3390/polym4010046

[37] Alfei S Schito AM. Positively charged polymers as promising devices against multidrug resistant Gram-negative bacteria: a review. Polymers. (2020) 12:1195. 10.3390/polym12051195 32456255 PMC7285334

[38] Poverenov E Klein M. Formation of contact active antimicrobial surfaces by covalent grafting of quaternary ammonium compounds. Colloids Surf B Biointerfaces. (2018) 169:195–205. 10.1016/j.colsurfb.2018.04.065 29778035

[39] Wang D Haapasalo M Gao Y Ma J Shen Y. Antibiofilm peptides against biofilms on titanium and hydroxyapatite surfaces. Bioactive Mater. (2018) 3:418–25. 10.1016/j.bioactmat.2018.06.002 30003180 PMC6039701

[40] Mi L Jiang S. Integrated antimicrobial and nonfouling zwitterionic polymers. Angew Chem Int Ed. (2014) 53:1746–54. 10.1002/anie.201304060 24446141

[41] Qi X Poernomo G Wang K Chen Y Chan-Park M-B Xu R. Covalent immobilization of nisin on multi-walled carbon nanotubes: superior antimicrobial and anti-biofilm properties. Nanoscale. (2011) 3:1874–80. 10.1039/c1nr10024f 21431164

[42] Mauriello G Ercolini D La Storia A Casaburi A Villani F. Development of polythene films for food packaging activated with an antilisterial bacteriocin from *Lactobacillus curvatus* 32Y. J Appl Microbiol. (2004) 97:314–22. 10.1111/j.1365-2672.2004.02299.x 15239697

[43] Hassan G Forsman N Wan X Keurulainen L Bimbo LM Johansson L-S. Dehydroabietylamine-based cellulose nanofibril films: a new class of sustainable biomaterials for highly efficient, broad-spectrum antimicrobial effects. ACS Sustainable Chem Eng. (2019) 7:5002–9. 10.1021/acssuschemeng.8b05658

[44] Hassan G Forsman N Wan X Keurulainen L Bimbo LM Stehl S. Non-leaching, highly biocompatible nanocellulose surfaces that efficiently resist fouling by bacteria in an artificial dermis model, ACS Appl Bio Mater. (2020) 3:4095–108. 10.1021/acsabm.0c00203 35025484

[45] Otzen D E. Biosurfactants and surfactants interacting with membranes and proteins: same but different? Biochim Biophys Acta Biomembr. (2017) 1859:639–49. 10.1016/j.bbamem.2016.09.024 27693345

[46] Richards S-J Isufi K Wilkins LE Lipecki J Fullam E Gibson MI. Multivalent antimicrobial polymer nanoparticles target mycobacteria and Gram-negative bacteria by distinct mechanisms. Biomacromolecules. (2018) 19:256–64. 10.1021/acs.biomac.7b01561 29195272 PMC5761047

[47] Pillai PP Kowalczyk B Kandere-Grzybowska K Borkowska M Grzybowski BA. Engineering Gram selectivity of mixed-charge gold nanoparticles by tuning the balance of surface charges. Angew Chem Int Ed. (2016) 55:8610–4. 10.1002/anie.201602965 27253138

[48] Das T Sharma PK Busscher HJ van der Mei HC Krom BP. Role of extracellular DNA in initial bacterial adhesion and surface aggregation. Appl Environ Microbiol. (2010) 76:3405–3408. 10.1128/AEM.03119-09 20363802 PMC2869138

[49] Saggu SK Jha G Mishra PC. Enzymatic degradation of biofilm by metalloprotease from *Microbacterium* sp. SKS10. Front Bioeng Biotechnol. (2019) 7:192. 10.3389/fbioe.2019.00192 31448272 PMC6692633

[50] Cheng Y Feng G Moraru CI. Micro- and nanotopography sensitive bacterial attachment mechanisms: A review. Front Microbiol. (2019) 10:191. 10.3389/fmicb.2019.00191 30846973 PMC6393346

[51] Hasan J Chatterjee K. Recent advances in engineering topography mediated antibacterial surfaces. Nanoscale. (2015) 7:15568–75. 10.1039/C5NR04156B 26372264 PMC4642214

[52] Epstein AK Wong T-S Belisle RA Boggs EM Aizenberg J. Liquid-infused structured surfaces with exceptional antibiofouling performance. Proc Natl Acad Sci USA. (2012) 109:13182–7. 10.1073/pnas.1201973109 22847405 PMC3421179

[53] Krasowska A Sigler K. How microorganisms use hydrophobicity and what does this mean for human needs? Front Cell Infect Microbiol. (2014) 4:112. 10.3389/fcimb.2014.00112 25191645 PMC4137226

[54] De-la-Pinta I Cobos M Ibarretxe J Montoya E Eraso E Guraya T. Effect of biomaterials hydrophobicity and roughness on biofilm development. J Mater Sci Mater Med. (2019) 30:77. 10.1007/s10856-019-6281-3 31218489

[55] Hook AL Chang C-Y Yang J Luckett J Cockayne A Atkinson S. Combinatorial discovery of polymers resistant to bacterial attachment. Nat Biotechnol. (2012) 30:868–75. 10.1038/nbt.2316 22885723 PMC3796337

[56] Im W Khalid S. Molecular simulations of Gram-negative bacterial membranes come of age. Annual Rev Phys Chem. (2020) 71:171–88. 10.1146/annurev-physchem-103019-033434 32070216

[57] Gumbart JC Beeby M Jensen GJ Roux B. *Escherichia coli* peptidoglycan structure and mechanics as predicted by atomic-scale simulations. PLoS Comput Biol. (2014) 10:e1003475. 10.1371/journal.pcbi.1003475 24586129 PMC3930494

[58] Gilmore BF Flynn PB O'Brien S Hickok N Freeman T Bourke P. Cold plasmas for biofilm control: opportunities and challenges. Trends Biotechnol. (2018) 36:627–38. 10.1016/j.tibtech.2018.03.007 29729997 PMC12168448

